# The anchor domain is critical for Piezo1 channel mechanosensitivity

**DOI:** 10.1080/19336950.2021.1923199

**Published:** 2021-05-11

**Authors:** Jinyuan Vero Li, Charles D Cox, Boris Martinac

**Affiliations:** a Molecular Cardiology and Biophysics Division, Victor Chang Cardiac Research Institute, Sydney, Australia; b St Vincent’s Clinical School, Faculty of Medicine, University of New South Wales, Sydney, Australia

**Keywords:** Mechanosensitive channels, force sensing, membrane tension

## Abstract

The mechanosensitive channel Piezo1 is a crucial membrane mechanosensor ubiquitously expressed in mammalian cell types. Critical to its function in mechanosensory transduction is its ability to change conformation in response to applied mechanical force. Here, we interrogate the role of the anchor domain in the mechanically induced gating of human Piezo1 channels. Using the insertion of glycine residues at each corner of the triangular-shaped anchor domain to decouple this domain we provide evidence that the anchor is important in Piezo1 mechano-gating. Insertion of two extra glycine residues between the anchor and the outer helix of human Piezo1 causes abrogated inactivation and reduced mechanosensitivity. Whereas inserting two glycine residues at the apex of the anchor domain at the conserved amino acid P2113 causes the channel to be more sensitive to membrane forces. Correlation of stretch sensitivity with the volume of the neighboring amino acid, natively a phenylalanine (F2114), suggests this is caused by removal of steric hindrance on the inner pore-lining helix. Smaller volume amino acids at this residue increase sensitivity whereas larger volume reduces mechanosensitivity. The combined data show that the anchor domain is a critical region for Piezo1-mediated force transduction.

## Introduction

Mechanosensitive (MS) channels are a ubiquitously expressed class of ion channels tasked with decoding mechanical cues [1]. They are expressed in all domains of life from single celled organisms (bacteria [2] and archaea [3]) to multicellular eukaryotes [4]. One of the central features of these channels is their ability to convert mechanical stimuli into a conformational change that underlies channel gating and ion flux. The first MS channels to be cloned were those found in bacteria [5,6]. These channels were shown to gate in response to membrane bilayer forces [6,7] and required no additional cytoskeletal or extracellular matrix components to function as mechanosensors. These original findings became known as the force-from-lipids principle whereby an MS channel converts membrane forces into conformational changes [8]. Work over the last decade has shown that many eukaryotic MS channels can also be gated in simplified systems containing only the channel and lipids [9–13]. While these systems represent a vast oversimplification of the mechanosensing landscape of a mammalian cell they do suggest that in many cases these structurally distinct ion channels can sense lipid bilayer forces. This raises two central questions; how do MS channels sense these forces and given the evolutionary conservation of such a mechanism are there common features between structurally distinct channels that enable bilayer-mediated mechanosensing?

Previous work on the evolutionary ancient mechanosensitive channel of large conductance (MscL) suggested that horizontal amphipathic helices may couple the channel to the bilayer [14–17]. In fact, multiple MS channels possess such structural entities and in some cases, these structures have been shown to be important in mechanosensing [18–20]. Moreover, other mechanosensing molecules including G-protein coupled receptors may also use a similar aamphipathic-helix-based mechanism for mechanosensing [21]. MscL and Piezo1 channels show no similarity in structure or sequence. MscL is a pentamer where each monomer is made of 136 amino acids [22] and Piezo1 is a trimer where each monomer is made of 2521 amino acids [20,23,24]. Piezo1 has a key role in cardiovascular physiology and can sense multiple types of mechanical stimuli including shear stress [25–27]. So, we asked could a horizontal helix also be important for Piezo1 channel mechano-gating when the structures of these channels are so diverse? Piezo1 channels possess a triskelion architecture where three propellers converge on a central pore flanked by a triangular structure termed the anchor domain [20,23,24]. The anchor domain contains three helices, one of which is a horizontal helix at the putative membrane interface.

Here we interrogated the role of the anchor domain in the mechano-gating of Piezo1 channels. We inserted two glycine residues in the corners of the anchor domain and found that the increased flexibility brought about by the glycine insertions differentially affects the mechanosensitivity of Piezo1. In particular, we found that inserting two glycine residues at the apex of the anchor domain at residue P2113 increased sensitivity. On inspection of this area, we speculated that this may occur through modification of the positioning of the highly conserved F2114 residue [28]. The F2114 side chain is arranged so that it directly points toward the pore-lining inner helix (IH) of the neighboring Piezo1 monomer. Using site-directed mutagenesis, we show that the sensitivity of the channel to membrane stretch correlates well with the volume of the amino acid in this position. Thus, F2114 may act to restrict IH movement and smaller residues make gating easier by removing this bulky residue. These data shed new light on the structural mechanism for mechano-gating in Piezo1 channels.

## Methods

### Cell lines

Piezo1^−/-^ HEK293T cells [29] were a gift from Dr Ardem Patapoutian (The Scripps Research Institute, La Jolla, CA, USA). Cells were not authenticated and were not listed in the database of commonly misidentified cell lines maintained by ICLAC (http://iclac.org) and NCBI Biosample (http://www.ncbi.nlm.nih.gov/biosample). Piezo1^−/-^ HEK293T cells were confirmed to be mycoplasma free.

### Mutagenesis

Site directed mutagenesis of human Piezo1 was undertaken using a custom protocol with the high-fidelity polymerase PfuUltra. Primers for mutagenesis are listed in Table 1.

**Table 1.** List of primers for site-directed mutagenesis of human (hP1) Piezo1

Primer	Sequence
hP1- F2113A Sense:	ccggctggtgccggccctggtggagctg
hP1- F2113A Anti-sense:	cagctccaccagggccggcaccagccgg
hP1- F2113 C Sense:	gctccaccaggcacggcaccagccg
hP1- F2113C Anti-sense:	cggctggtgccgtgcctggtggagc
hP1- F2113G Sense:	gttccggctggtgccgggcctggtggagctg
hP1- F2113G Anti-sense:	gttccggctggtgccgggcctggtggagctg
hP1- F2113T Sense:	ccggctggtgccgaccctggtggagctg
hP1- F2113T Anti-sense:	cagctccaccagggtcggcaccagccgg
hP1- F2113Y Sense:	gcagctccaccagatacggcaccagccg
hP1- F2113Y Anti-sense:	cggctggtgccgtatctggtggagctgc
hP1- G2163 + 2 G Sense:	tacccgcagcccaaagggggtggacagaagaagaagaagatc
hP1- G2163 + 2 G Anti-sense:	gatcttcttcttcttctgtccaccccctttgggctgcgggta
hP1- P2113 + 2 G Sense:	ttccggctggtgccgggtggattcctggtggagctg
hP1- P2113 + 2 G Anti-sense:	cagctccaccaggaatccacccggcaccagccggaa
hP1- T2128 + 2 G Sense:	gtgtggacggacaccggtggaacgctgtccctgtcc
hP1- T2128 + 2 G Anti-sense:	ggacagggacagcgttccaccggtgtccgtccacac

### Western blotting

Cells were cultured in Dulbecco’s modified Eagle medium (DMEM; Sigma-Aldrich, St. Louis, MO, USA) supplemented with 10% v/v fetal bovine serum (ThermoFisher Scientific, Waltham, MA, USA) and incubated at 37°C with 5% CO_2._ IRES EGFP WT and mutant human Piezo1 cloned from HEK cells [30] were transfected into HEK293T cells, using Lipofectamine 3000 transfection reagent (ThermoFisher Scientific) with 500 ng of DNA. The medium was changed 24 h after transfection. Cells were harvested 72 h after transfection and solubilized in radio-immunoprecipitation assay buffer (RIPA) [Tris 10 mM, ethylenediaminetetraacetic acid (EDTA) 1 mM, NaCl 140 mM, in (% w/v): Sodium deoxycholate 0.1, SDS 0.1, Triton X-100 1.0, pH 7.2] supplemented with 1 × EDTA-free protease inhibitor cocktail tablets (Sigma-Aldrich), 1 mM (phenylmethylsulfonyl fluoride) PMSF, 2 mM tris(2-carboxyethyl)phosphine (TCEP), and 1 mM *N*-ethylmaleimide (NEM) on ice for 10 min. Cell lysates were cleared by centrifugation at 13,000 × g at 4°C for 20 min.

For Western blot analysis the human Piezo1 channel was probed using a mouse monoclonal anti-Piezo1 antibody (Cat# NBP2-75617, Novus Biologicals, Centennial, CO, USA; 1:1,000 dilution) and loading controls determined using mouse anti-α-actinin antibody (Abcam; 1:5,000 dilution). Blots were visualized using an anti-mouse IRDye800 at 1:20,000 (Li-Cor) to enable quantification with the LI-COR Odyssey system (LI-COR Biotechnology, Lincoln, NE, USA). Image studio (LI-COR Biotechnology) was used to generate representative Western blot images.

### Electrophysiology

Transiently transfected Piezo1^−/-^ HEK293T cells were plated on 35 mm dishes for patch clamp analysis. The extracellular solution for cell-attached patches contained high K^+^ to zero the membrane potential; it consisted of 90 mM potassium aspartate, 50 mM KCl, 1 mM MgCl_2_ and 10 mM HEPES (pH 7.2) adjusted with 5 M KOH. The pipette solution contained 140 mM CsCl with 10 mM HEPES (pH 7.2) adjusted with CsOH. Ethylene glycol-bis(β-aminoethyl ether)-*N,N,N*′,*N*′-tetraacetic acid (EGTA) was added to control levels of free pipette (extracellular) Ca^2+^ using the online EGTA calculator – Ca-EGTA Calculator TS v1.3 – Maxchelator. Negative pressure was applied to patch pipettes using a High-Speed Pressure Clamp-1 (ALA Scientific Instruments, Farmingdale, NY, USA) and recorded in millimeters of mercury (mmHg) using a piezoelectric pressure transducer (WPI, Sarasota, FL, USA). Borosilicate glass pipettes (Sigma-Aldrich) were pulled with a vertical pipette puller (PP-83, Narashige, Tokyo, Japan) to produce electrodes with a resistance of 1.6–2.4 MΩ. Single-channel Piezo1 currents were amplified using an AxoPatch 200B amplifier (Axon Instruments, Union City, CA, USA), and data were sampled at a rate of 10 kHz with 1 kHz filtration and analyzed using pCLAMP10 software (Axon Instruments). The Boltzmann distribution function was used to describe the dependence of mesoscopic Piezo1 channel currents and open probability, respectively, on the negative pressure applied to patch pipettes. Boltzmann plots were obtained by fitting open probability *P*_o_∼*I*/*I*_max_ versus negative pressure using *P*_o_/(1–*P*_o_) = exp [*α* (*P*–*P*_1/2_)], where *P* is the negative pressure (suction) in mm Hg, *P*_1/2_ is the negative pressure at which *P*_o_ = 0.5, and *α* (mm Hg)^−^[1] is the slope of the plot of ln [*P*_o_/(1–*P*_o_)] versus (*P*–*P*_1/2_), reflecting the channels’ mechanosensitivity. Peak currents were measured using Clampfit (Axon Instruments).

## Results

### Insertion of glycine residues (+2 G) within the anchor domain modifies Piezo1 function

**Figure 1.**
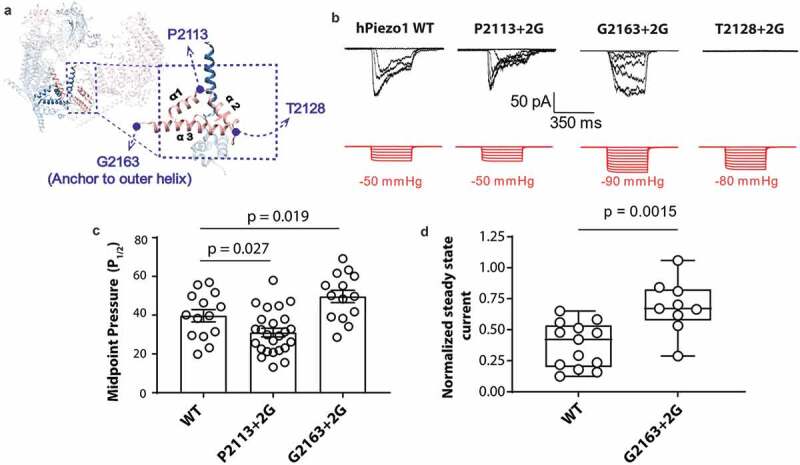
**Glycine insertions within the anchor domain influence Piezo1 channel mechanosensitivity**. (a) Representative electrophysiological traces for WT human Piezo1 (hPiezo1) and the P2113 + 2 G, G2163 + 2 G and T2128 + 2 G mutants. Cell-attached configuration with current shown in black and pressure trace in red, recorded at +60 mV pipette potential. (c) Quantification of midpoint pressure threshold (P_1/2_) for WT, P2113 + 2 G and G2163 + 2 G determined by fitting pressure response curves with Boltzmann fit. Data is displayed as mean ± S.E.M. (p – value determined using One-way ANOVA with Sidak’s post hoc test). (d) Normalized steady state current of the G2163 + 2 G mutant showing a significantly higher value than WT indicative of abrogated inactivation. Normalized steady state current was measured for WT and G2163 + 2 G at the first pressure that induced currents at or above the P_1/2_ to normalize for pressure application. Data is displayed as a maximum to minimum box and whisker plot with all data points shown (p – value determined using T-test)

To interrogate the role of the anchor domain in the mechanosensing of Piezo1 we took a similar approach to work on the structurally unrelated bacterial MS channel MscL [15]. Namely, we tried to decouple the anchor domain from the pore of Piezo1 using the insertion of glycine residues. The anchor domain forms a triangle of three helices, so we inserted two glycine residues at each corner of the anchor domain at position P2113, T2128 and G2163 which we named P2113 + 2 G, T2128 + 2 G and G2163 + 2 G, respectively (Figure 1a). These glycine residues increase conformational freedom and if the anchor domain was an important structure for mechanosensitivity, we hypothesized that these mutations should change the sensitivity of Piezo1 to membrane tension. To test this, we characterized these three mutants using cell-attached patch-clamp recordings in a Piezo1^−/-^ HEK293T cell line and compared their response to wild-type (WT) human Piezo1 (hPiezo1). Specifically, we applied negative hydrostatic pressure as a square wave pulse of 350 ms duration. On initial interrogation, we found that both P2113 + 2 G and G2163 + 2 G gave stretch-activated currents when expressed in Piezo1^−/-^ HEK293T cell line (Figure 1b). Interestingly, we found that P2113 + 2 G and G2163 + 2 G have the opposite impact on Piezo1 mechanosensitivity (Figure 1c). P2113 + 2 G causes an increase in the channel mechanosensitivity reflected in reduction of the P_1/2_ in response to stretch and a corresponding leftward shift in the pressure response curve. In contrast,G2163 + 2 G caused a rightward shift in the pressure response curve indicating reduced sensitivity (indicated by an increase in P_1/2_) to mechanical force in the form of membrane stretch (Figure 1c). The G2163 + 2 G also displayed slower inactivation which was quantified using the normalized state current (Figure 1b, d) [30,31].

### T2128 + 2 G is trafficking defective

**Figure 2.**
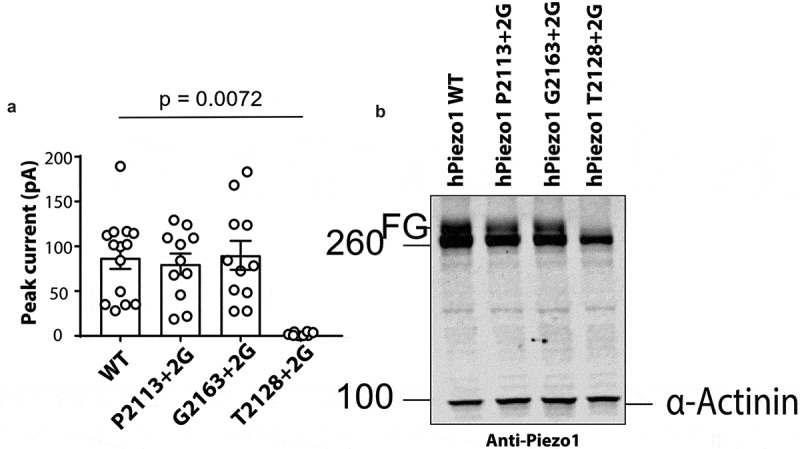
**Glycine insertion (+2 G) at position T2128 results in a trafficking defect**. (a) Peak currents elicited per cell-attached patch comparing WT hPiezo1 to the T2128 + 2 G mutant at +60 mV pipette potential (p – value determined using T-test. (b) Representative western blot showing WT, P2113 + 2 G, G2163 + 2 G and T2128 + 2 G (FG – Fully glycosylated), (n = 5)

We noted that the T2128 + 2 G mutant gave almost no current in response to stretch (Figure 1b & Figure 2a). This can be seen in the representative electrophysiological trace shown in Figure 1b and is quantified in Figure 2b. The peak current elicited per patch was 2.08 ± 0.7 pA (n = 8) for the T2128 + 2 G mutant compared to the WT current of 87.05 ± 12.2 pA (n = 14). This mutation could have impaired stretch induced channel gating, or it could be misfolded and not traffic correctly to the surface. To understand why this mutation lacks function we used western blotting. We have previously shown that human Piezo1 undergoes significant N-linked glycosylation and that the fully glycosylated version of the protein represents the major pool of the surface expressed mature Piezo1^32^. So we expressed wild-type P2113 + 2 G, T2128 + 2 G and G2163 + 2 G in Piezo1^−/-^ HEK293T and probed their expression using a primary monoclonal Piezo1 antibody [32]. A representative western blot clearly showed that WT, P2113 + 2 G and G2163 + 2 G all run as a doublet with the upper band representing the fully glycosylated (FG) protein. In contrast while T2128 + 2 G does express the full-length protein (~286 kDa), the upper band of the doublet is completely missing indicative of a trafficking defect. As a result, we did not further characterize T2128 + 2 G.

### F2114 is important for Piezo1 channel mechanosensitivity

**Figure 3.**
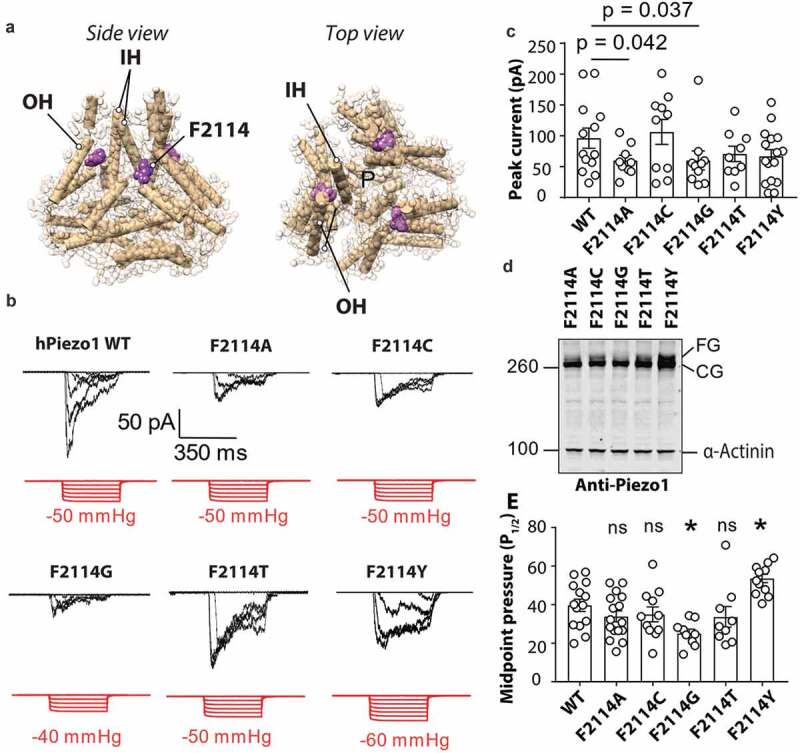
**Mutations at position F2114 in human Piezo1 influence channel sensitivity to mechanical force**. (a) Side and top view of Piezo1 structure showing the inner helix (IH) outer helix (OH) and the position of F2114 (purple). Note that the F2114 residue is in very close proximity to the IH which lines the pore (denoted – P). (b) Representative electrophysiological traces for WT, F2114G, F2114A, F2114C, F2114T and F2114Y. Cell-attached configuration with current shown in black and pressure trace in red, recorded at +60 mV pipette potential. (c) Comparison of peak current elicited per patch for WT hPiezo1 and F2114 mutations (p – value determined using One-way ANOVA with Sidak’s multiple comparison test, only significant comparisons with WT illustrated). (d) Representative blot showing Piezo1 protein levels for transiently transfected mutants F2114G, F2114A, F2114C, F2114T and F2114Y after 72 h expression (n = 4). Note the lower level of the upper band with F2114G (FG – fully glycosylated, CG – core glycosylated). (e) Quantification of midpoint pressure threshold (P_1/2_) in mmHg for WT, F2114G, F2114A, F2114C, F2114T and F2114Y determined by fitting pressure response curves with Boltzmann fit. (ns – not significant, * – significant with p = 0.0232 F2114 and 0.0136, determined using One-way ANOVA with Sidak’s multiple comparison test). Data is displayed as mean ± S.E.M

Intrigued by the fact that P2113 + 2 G was more sensitive to mechanical force we looked at a potential mechanism for how this may occur. The adjacent residue F2114 points directly toward the inner helix (IH) that lines the pore of Piezo1 (Figure 3a). Due to the domain swapped architecture of Piezo1 the F2114 from one monomer comes to within proximity of the pore-lining inner helix on the neighboring monomer. To test whether the increased sensitivity observed for the P2113 + 2 G mutant may be explained by a change in conformation of F2114 we generated five mutations at this site mutating the native phenylalanine to glycine, alanine, cysteine, threonine, and tyrosine. These mutants were then expressed in Piezo1^−/-^ HEK293T and their response to membrane stretch was determined. All five mutations produced stretch-activated currents in response to negative hydrostatic pressure (Figure 3b). The peak currents per patch were slightly smaller for F2114G (60.18 ± 15.4 pA) and F2114A (60.10 ± 8.3 pA) (Figure 3c). We then looked at the expression of each of the F2114 mutations using western blotting and the same monoclonal anti-Piezo1 antibody used in Figure 2. We saw that compared to the other four F2114 mutations (A, C, T, Y) the F2114G in particular, but also the F2114A, has a lower intensity upper band (the fully glycosylated version) consistent with previous studies (Figure 3d) [32]. We then compared the mechanosensitivity of all the variants at the 2114 position (Figure 3e). The midpoint threshold (P_1/2_) for each mutation was calculated by fitting the pressure response curve with a Boltzmann fit with the cumulative data displayed in Figure 3e. F2114G had a lower P_1/2_ (25.12 ± 2.1 mmHg) than WT Piezo1 whereas the F2114Y mutation had a larger P_1/2_ (53.61 ± 2.2mmHg).

### Volume of F2114 correlates with Piezo1 channel mechanosensitivity

**Figure 4.**
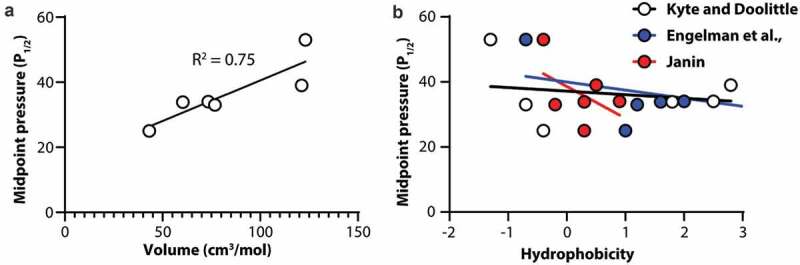
**Correlation of Piezo1 channel mechanosensitivity with amino acid volume and hydrophobicity at amino acid position 2114**. (a) Midpoint pressure (P_1/2_) plotted against amino acid volume. (b) Midpoint pressure (P_1/2_) plotted against amino acid hydrophobicity using three different scales. In all cases solid lines represent linear regression

To understand what specific characteristics of the side chain at position F2114 were determining the mechanosensitivity of Piezo1 we correlated the P_1/2_ of activation as a measure of mechanosensitivity of the channel with both the volume of the side chain in cm^3^/mol [33] and the hydrophobicity of the amino acid [34–36]. We identified that the P_1/2_ of the channel shows a robust correlation with amino acid volume (Figure 4a). Using linear regression, the R^2^ fit value is 0.75 denoting a good correlation between volume and P_1/2_. In contrast when we correlate amino acid hydrophobicity, using three different hydrophobicity scales, with P_1/2_ as a measure of channel mechanosensitivity, there is little to no correlations in all three cases (Kyte and Doolittle; R^2^ = 0.04, Engelman et al.; R^2^ = 0.14, Janin; R^2^ = 0.24).

## Discussion

MS channels are critical to the physiology of all organisms [1]. They display a vast divergence in structure suggesting ion channel mechanosensitivity has arisen multiple times during evolution [37,38]. Despite the lack of structural similarity, many of the MS channel families discovered to date respond to membrane forces [4,10,12,13,39]. This may suggest that a common structural feature, much like the signature sequence for Ca^2+^ binding or K^+^/Na^+^ channel selectivity, may be present that underlies ion channel mechanosensitivity via the force-from-lipids concept [7].

We have previously suggested that horizontal, bilayer coupling, amphipathic helices may represent the lowest common denominator driving bilayer mediated ion channel mechanosensitivity [14–17,40]. In the present study, we interrogated the anchor domain of the Piezo1 MS channel that harbors a horizontal helix to determine whether this region is indeed involved in mechanosensitivity. We took an approach similar to that used in the less structurally embellished, evolutionary ancient MS channel MscL [14,15]. Specifically, we inserted two flexible glycine residues within the anchor domain to attempt to decouple the anchor domain from the pore to see if this region is important in mechano-gating.

We found that introducing flexibility between the anchor domain and the outer helix (G2163 + 2 G) had a dual effect. Firstly, it reduced channel inactivation. This is consistent with this region, being important for inactivation including a string of lysine residues in this area [31]. A mutant lacking all four lysine residues was previously linked to the hereditary anemia xerocytosis [41]. Secondly, G2163 + 2 G was harder to open as indicated by a rightward shift in the pressure response curve and a higher midpoint threshold. This suggests that the link between the anchor and the outer helix is important for conveying force as the flexibility induced by the two glycines reduced mechanosensitivity.

We also highlighted that the insertion of two glycine residues at T2128 results in a nonfunctional channel. This is consistent with our previous work showing that mutants of Piezo1 which lack the fully glycosylated version of the channel have aberrant membrane targeting. Thus, we concluded that T2128 + 2 G is likely a trafficking defective mutant and we did not further characterize this mutation.

In the case of two glycine residues inserted at the apex of the anchor domain at residue P2113 we observed that the channel became more sensitive to mechanical force evidenced by a leftward shift in the pressure response curve and a lower P_1/2_. To understand why flexibility introduced at the apex of the anchor modified mechanosensitivity, we focussed on the neighboring residue F2114. This bulky residue is highly conserved in Piezo1 homologues [28] and sits in close proximity to the pore-lining inner helix of the adjacent monomer. We mutated this residue to amino acids with different size and polarity to see how this influenced mechanosensitive gating in Piezo1. The volume of the residue present at this position correlated well with the mechanosensitivity of Piezo1. As the volume of the residue present became larger, the P_1/2_ of the Piezo1 channel also increased. This suggests that rather than pulling on the IH, the F2114 residue applies a “break” or resistive force on the IH, and making it smaller, for example by replacing it with glycine or by introducing more conformational freedom as in the P2113 + 2 G mutant, reduces the force necessary for gating. These data combined illustrate the anchor domain is critical for Piezo1 mechanosensing.

If the anchor domain alone was necessary for mechanosensitivity, then one might assume that the distal C-terminus may exhibit mechanosensitivity. In fact, current data suggest that the full propellers are needed for mechanosensing in Piezo1 channels [42,43]. This means the anchor domain alone is unlikely to recapitulate the mechano-gating of Piezo1^42^[43]. In addition, many other regions including the local curvature and “footprint” [23,44] of the Piezo1 channel are essential for mechanosensing [45]. This could include the importance of cytoskeletal [46,47] or extracellular-matrix-based molecular tethers (possibly N-glycans [32]) that work in unison with bilayer forces [38]. In conclusion, our data suggest that the anchor domain, although not the only, is a critical force conveying structure within the MS channel Piezo1.
